# Strategies for Delivering Mental Health Services in Response to Global Climate Change: A Narrative Review

**DOI:** 10.3390/ijerph17228562

**Published:** 2020-11-18

**Authors:** Lawrence A. Palinkas, Meaghan L. O’Donnell, Winnie Lau, Marleen Wong

**Affiliations:** 1 Suzanne Dworak-Peck School of Social Work, University of Southern California, Los Angeles, CA 90089-0411, USA; marleenw@usc.edu; 2 Phoenix Australia Centre for Posttraumatic Mental Health and Department of Psychiatry, The University of Melbourne, Carlton, VIC 3053, Australia; mod@unimelb.edu.au (M.L.O.); wlau@unimelb.edu.au (W.L.)

**Keywords:** mental health services, climate change, disasters, trauma, prevention, treatment

## Abstract

This narrative review examined strategies for preparedness and response to mental health impacts of three forms of climate change from a services perspective: (1) acute and extreme weather events such as hurricanes, floods, and wildfires, (2) sub-acute or long-term events such as droughts and heatwaves; and (3) the prospect of long-term and permanent changes, including higher temperatures, rising sea levels, and an uninhabitable physical environment. Strategies for acute events included development and implementation of programs and practices for monitoring and treating mental health problems and strengthening individual and community resilience, training of community health workers to deliver services, and conducting inventories of available resources and assessments of at-risk populations. Additional strategies for sub-acute changes included advocacy for mitigation policies and programs and adaptation of guidelines and interventions to address the secondary impacts of sub-acute events, such as threats to livelihood, health and well-being, population displacement, environmental degradation, and civil conflict. Strategies for long-lasting changes included the implementation of evidence-based risk communication interventions that address the existing and potential threat of climate change, promoting the mental health benefits of environmental conservation, and promoting psychological growth and resilience.

## 1. Introduction

The mental health impacts of global climate change have been well-documented [1,2,3,4,5,6,7,8,9,10,11]. These impacts will be widespread, profound, and cumulative [3,6] and include elevated rates of anxiety and mood disorders, acute stress reactions and posttraumatic stress disorders, sleep disruption, suicide and suicidal ideation, substance use disorders, and a decreased sense of self and identity from loss of place and connection to the environment [1,2,6,7,8,10,11,12,13,14,15,16,17,18,19,20]. Indeed, numerous studies have linked three specific forms of climate change to mental health impacts: (1) acute and extreme weather events (EWEs) and natural disasters, such as hurricanes [8,12], floods [13,14], wildfires [15], and heat waves lasting for days or weeks [16,17]; (2) sub-acute weather events lasting for months or years such as droughts [1,2,18,19] and longer-duration heat waves [1,2,20]; and (3) and a permanently altered and potentially uninhabitable physical environment that is either too dry or too wet, but ultimately too hot [1,2,4,5,7,9,10]. Each form of climate change can contribute to an increase in mental health problems that are directly linked to property damage, loss of income and employment opportunities and reduced economic productivity [1,2,19,21], threats to health and well-being associated with EWE injuries and deaths, spread of infectious diseases and respiratory illnesses, and heat-related stress [1,2,11]; population displacement [22], damage to the natural environment [1,2,3,7,19], and civil conflicts and episodes of communal violence [23,24,25]. Moreover, the distinctions between the three forms of climate change and their mental health impacts are beginning to blur as acute events like wildfires and hurricanes that appear year after year with increasing intensity in the same regions (e.g., wildfires in Australia and the Western United States, hurricanes in the Gulf of Mexico) take on the characteristics of long-term sub-acute events such as droughts and generate the same forms of anxiety as long-term climate change [26,27].

However, most of the research to date has focused on the epidemiology of these impacts. Although policy and practice implications of these impacts are frequently mentioned, they relate primarily to EWE’s like hurricanes, floods, wildfires, and heatwaves. A comprehensive services response to these impacts should also consider longer-term events such as prolonged droughts, sea-level rise and coastal erosion, and constant exposure to high temperatures.

### Aims

In this narrative review, different types of mental health impacts and strategies for preparedness and response to these impacts were examined from a services perspective. The aim of this review was to identify and describe the different types and characteristics of evidence-informed and evidence-based interventions for the prevention and treatment of mental and behavioral health problems associated with the three-forms of climate change events, assess potential barriers and facilitators to their implementation, and to examine the potential role of specialist and non-specialist mental health service providers in broader social, political, and public health approaches to addressing these problems through advocacy, mitigation, and adaptation. We used the continuum of care for mental health services developed by the Institute of Medicine [28] to serve as a framework for outlining the range of services necessary for addressing the mental health impacts of climate-related events. This framework outlines three categories of interventions that fall into the following categories: prevention, treatment, and maintenance. In this review, we focus on a stepped care strategy that prioritizes universal, selective, and indicated services aimed at prevention and standard treatments for known disorders.

## 2. Methods

A narrative literature review was conducted on mental health services and interventions that have been proposed and/or evaluated for effectiveness in responding to the mental health impacts associated with the three forms of climate-related changes in the environment (acute, sub-acute, and long-lasting). This method was selected because the literature to date has focused almost exclusively on the epidemiology of climate-related mental health problems, and the few studies that focus specifically on the delivery of services or interventions to prevent or treat such problems are not yet suitable for scoping or systematic reviews [29]. Nevertheless, this review followed a systematic data collection process using a defined set of criteria and standardized data extraction tools, which are recommended to minimize bias and increase the quality of narrative reviews [29].

### 2.1. Eligibility Criteria

The inclusion and exclusion criteria for the selection of empirical studies of mental health and psychosocial interventions based on the PICOS (participants, interventions, comparisons, outcomes, and study design) model are listed in Table 1 below. To be included, interventions had to focus on prevention or treatment of a mental health or psychosocial problem that had been proposed or evaluated for effectiveness in response to a specific form of environmental climate-related change (hurricanes, typhoons, flooding, wildfires, droughts, heatwaves, sea-level rise, coastal erosion) or had been proposed for such use based on empirical studies conducted in other types of disasters (natural, manmade), acts of terrorism, or civil conflict. As this is an exploratory review, no bias assessment was conducted.

### 2.2. Data Collection

Peer-reviewed papers and additional sources (government documents) were identified by the first author using the following strategies: PubMed and PsycINFO searches of the English language peer-reviewed literature published from 1 January, 2000 through 30 October, 2020 with the following keywords in the title and/or abstract: “disaster mental health” OR “climate change” AND “services” OR “interventions”, AND “effectiveness” AND “hurricanes” OR “floods” OR “wildfires” OR “droughts” OR “heatwaves” OR “sea-level rise” OR “coastal erosion” OR “ecoanxiety”. Resources were filtered using abstracts (where available), before evaluating full texts, and the included references were selected based on their relevance to the aim of this review. The reference section of each examined article was also reviewed to identify other potential studies. Other literature included disaster preparedness and recovery guidelines.

### 2.3. Ratings of Scientific Evidence

Building on a scale used by the California Evidence-Based Clearinghouse for Child Welfare (CEBC) [34] for evaluating the quality of scientific evidence supporting interventions for children, youth, and families, empirical studies of the effectiveness of specific interventions for prevention and treatment of mental health problems arising from the three forms of climate-related changes in the environment were rated using a five-point scale. Interventions rated as well supported by the research evidence were assigned the best rating of 1, if they met the following six criteria:

1. Multiple site replication and follow-up: At least two rigorous randomized controlled trials (RCTs) with nonoverlapping analytic samples that were carried out in usual care or practice settings have found the program to be superior to an appropriate comparison program on outcomes specified in the criteria for that particular topic area. In at least one of these RCTs, the program has shown to have a sustained effect at least one year beyond the end of treatment, when compared to a control group. The RCTs have been reported in published, peer-reviewed literature;
2. Outcome measures must be reliable and valid, and administered consistently and accurately across all subjects;
3. The overall weight of the published, peer-reviewed research evidence supports the benefit of the program for the outcomes specified in the criteria for that particular topic area.
4. There is no case data suggesting a risk of harm that: (a) was probably caused by the program and (b) was severe or frequent;
5. There is no legal or empirical basis suggesting that, compared to its likely benefits, the program constitutes a risk of harm to those receiving it;
6. The program has a book, manual, and/or other available writings that specify components of the service and describe how to administer it.

Along with criteria 2–6 of the interventions rated as well supported, interventions rated as supported by the research evidence were assigned a rating of 2 if the criteria for randomized controlled trial and follow-up was satisfied by at least one rigorous RCT that had found the program to be superior to an appropriate comparison program on outcomes specified in the criteria for that particular topic area; and if the program was shown to have a sustained effect of at least six months beyond the end of treatment, when compared to a control group. Interventions rated as promising were assigned a rating of 3 if there was at least one study utilizing some form of control (e.g., untreated group, placebo group, matched wait list study) and was reported in published, peer-reviewed literature as having: (a) Established the program’s benefit over the control on outcomes specified in the criteria for that particular topic area; (b) Found it to be comparable on outcomes specified in the criteria for that particular topic area to a program rated a 1, 2, or 3 on this rating scale in the same topic area; or (c) Found it to be superior on outcomes specified in the criteria for that particular topic area to an appropriate comparison program. Interventions rated as possibly effective were assigned a rating of 4 if they provided evidence of significant improvements in symptoms in pre-post comparisons. Finally, interventions were not rated (NR) if the program did not have any published, peer-reviewed study utilizing some form of control (e.g., untreated condition or group, placebo group, matched wait list study) that had established the program’s benefit over the control on outcomes specified in the criteria for that particular topic area, or found it to be comparable to or better than an appropriate comparison program on outcomes specified in the criteria for that particular topic area

## 3. Results

**Figure 1:**
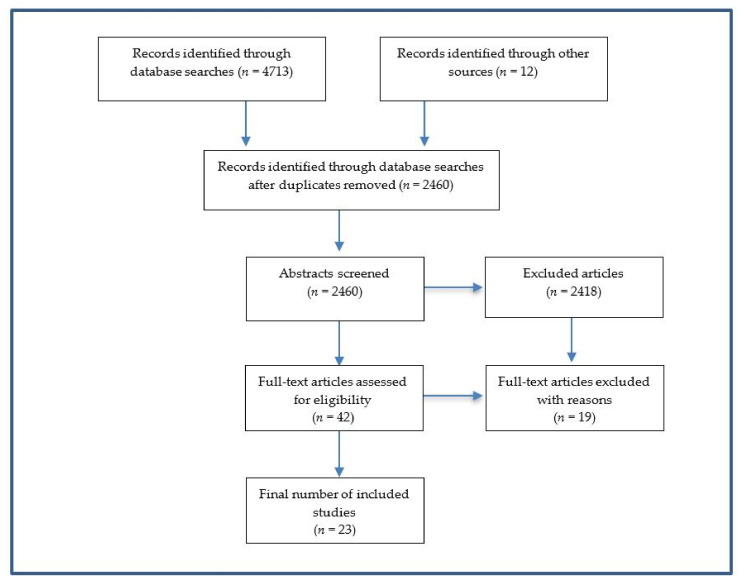
Screening and selection of eligible studies.

As outlined in Figure 1 a total of 2460 abstracts were reviewed, with 23 studies included in the final review. Table 2 presents the findings of the review with interventions categorized into the IOM categories. All three forms of climate change were associated with an increase in prevalence and severity of mental health problems such as depression, anxiety, posttraumatic stress disorder, excessive drug and alcohol use, and suicidal behavior, often accompanied by long-term psychosocial impairment. The prevalence, severity, and duration of these outcomes also vary by type and onset of the event. For instance, posttraumatic stress disorder was more prominent during acute events such as hurricanes and wildfires than during a sub-acute event or long-term changes in the environment [2,8,12,13,14,15]. Suicide has been linked to exposure to sub-acute events like droughts [18,19], while increased anxiety and mood disorders have been associated with long-term changes in the physical environment, especially among youth and young adults [35,36], as well as acute and sub-acute events. While these risk factors across events are important, mental health services need to be agile and responsive to the variable mental health effects that disaster survivors may experience. As such, training and guidance in the assessment and treatment of climate-related mental health problems and the development and implementation of evidence-based or informed treatments and prevention practices [37] is imperative. However, addressing these problems also requires a policy context that addresses the political and social aspects of disaster risk reduction. A comprehensive effort to prevent and treat these problems would also involve advocacy for climate change mitigation by service providers [3,10] and others that is consistent with national and international policies such as the United Nations Sustainable Development Goals (SDGs), especially Goals 3 (Good Health and Well-Being) and 13 (Climate Action) [38], the Sendai Framework for Disaster Risk Reduction 2015–2030 [32], and the United Nations Framework Convention on Climate Change (UNFCCC or Paris Agreement) [39].

### 3.1. Mental Health Services for Acute Events

Delivery of effective mental health services in the aftermath of acute events calls for coordinated planning and preparedness before the occurrence of such events and the use of evidence-based interventions afterward. Such services will require the development and implementation of effective and sustainable programs and practices for monitoring and treating mental health problems and strengthening individual and community resilience, training of teachers and community health workers to deliver mental health services, and conducting inventories of available resources and assessments of at-risk populations [3,10].

### 3.2. Guideline and Intervention Development and Implementation

There have been numerous guidelines for responding to the mental health needs of survivors of EWEs and other disasters. The mental health needs of affected people were first seen in the Sendai Framework for Disaster Risk Reduction (DRR) 2015–2030 [32], which provided recommendations to enhance recovery schemes to provide psychosocial support and mental health services for all people in need at the national level for disaster risk reduction. The European Network for Traumatic Stress (TENTS) [40] established guidelines for planning, preparation, and management, as well as general and specific response protocols. Another set of guidelines, developed by the World Health Organization (WHO) [13,30] and the Inter-Agency Standing Committee (IASC) [31] included both clinical mental health interventions and basic, non-clinical psychological support interventions that the IASC referred to as “Mental Health and Psychosocial Support” (MHPSS) [31]. It is generally the case that only a small proportion of disaster-affected populations require intensive treatment delivered by trained professionals. However, most disaster survivors would benefit from a range of lower intensity interventions that can help reduce distress [14].

Included in these guidelines are recommendations for a stepped-care approach to mental health service delivery to support different levels of interventions depending on the timing of the disaster and level of distress [10,41]. These stepped care approaches comprise universal, selective, indicated, and standard clinical intervention components [27]. Universal strategies target all people exposed to the disaster and are usually implemented immediately after disaster. Selective strategies target disaster survivors who are at increased risk of developing a mental, emotional or behavioral disorder based on environmental, biological, psychological or social characteristics. Indicated approaches target subgroups of those ‘at risk’ who are predisposed to or exhibit detectable signs and symptoms that are associated with a clinically significant disorder and are used later in the recovery process. Selective and indicated approaches largely focus on resilience building and psychosocial recovery. These approaches can be stepped up to clinical interventions using evidence-based treatments (EBTs) administered by trained mental health professionals if necessary. Often universal, selective and indicated interventions are delivered by non-specialists as a form of “task-shifting”, recommended by the WHO [42] for use particularly in settings, including communities devastated by natural disasters, where a shortage of specialists restricts access to standard treatments. Task-shifting delivery of mental health services to non-specialists like community health workers, teachers, or spiritual leaders, has been demonstrated to be effective in increasing access to and acceptability of services and treating a range of mental health disorders in low resource settings [43,44,45,46]. Task shifting can also serve to increase the autonomy and capacity of communities to deliver sustainable interventions. For instance, the Great East Japan Earthquake of 2011 led to the use of task-shifting with the establishment of Disaster Psychiatric Assistance Teams (DPATs) to address the mental health needs of people with pre-existing psychiatric conditions and first responders as well as educate and supervise first responders in the use of universal psychosocial interventions [47].

A list of mental health and psychosocial interventions that have been proposed for use in preventing and treating mental health problems associated with the three forms of climate-related changes in the environment is provided in Table 3 below. Ratings of scientific evidence were based on their use in addressing mental health problems associated with acute climate events, sub-acute events, and long-lasting events only. As indicated by the table, some interventions have yet to be investigated in the context of these events and were thus assigned a rating of “not rated” (NR).

Psychological First Aid (PFA) [48] is an evidence-informed universal approach in mental health services delivery that includes the provision of information, comfort, emotional and instrumental support to those exposed to a traumatic and life-threatening event, with assistance provided in a modular and stepwise fashion tailored to the person’s needs. PFA is designed to be used by first responders and other non-mental health specialists for the purpose of assessing the level of distress and need for more intensive services in the aftermath of a traumatic event like a natural disaster, facilitating connections to social support networks, and fostering adaptive functioning and coping [49]. One study conducted 20 months after Hurricane Katrina with 5–15 year-old displaced children reported a statistically significant reduction in PTSD symptoms [50]. However, although it is considered to be an “evidence-informed” intervention [51], reviews of PFA to date have found no evidence of its effectiveness in providing psychosocial support to disaster and trauma survivors [52].

Service providers also play an active role in fostering community resilience through the implementation of universal and selective interventions designed to prevent adverse mental health outcomes in at risk populations [3,53]. One example of such an intervention is the Strengthening Families Program (SFP) [54], a family skills training program designed to increase resilience and reduce risk factors for behavioral, emotional, academic, and social problems in 3–16 year-olds. Although a culturally adapted version of SFP was found to be effective in reducing externalizing problems in a randomized controlled trial with 7–15-year-old Burmese refugee youths in Thailand [55], there have been no studies to date evaluating its effectiveness in survivors of acute weather events. On the other hand, using an RCT design, the group-based, Integrated Mental Health and Disaster Preparedness intervention was tested in three earthquake-exposed and flood-prone communities in Haiti and found to significantly increase peer-based help-giving and help-seeking, as well as reduce symptoms of PTSD, depression, anxiety and functional impairment [56].

Non-mental health specialists also play a critical role in the delivery of indicated stepped-care approaches to the delivery of mental health services. These indicated interventions are often brief and low in intensity. One form of indicated intervention is Problem Management Plus (PM+)*,* designed for use in communities affected by adversity [57]. Developed as part of the WHO Mental Health Gap Action Programme (mhGAP) [42], PM+ is available in both individual (Individual PM+) and group (Group PM+) versions. However, despite evidence of its effectiveness in low-income countries exposed to adversity [58,59,60], it has not been explicitly evaluated following a natural disaster. Skills for Psychological Recovery (SPR) is another indicated stepped approach designed for the treatment of post-disaster mental health problems and is consistent with empirically supported principles following a disaster [61]. Although it has been demonstrated to be acceptable to clinicians and cost-effective in simulated agent-based models [40], its effectiveness has yet to be evaluated using randomized controlled trials.

Skills for Life Adjustment and Resilience (SOLAR) is another indicated intervention that has been intentionally designed to mitigate poor psychosocial adjustment and distress after a natural disaster and other traumatic events [62,63]. Importantly, SOLAR targets key mechanisms that are regarded as central to trauma recovery, including emotional processing of the traumatic event. It is deliverable by non-mental health professionals after a short period of training and can be delivered to individuals or groups. This emphasis on task-shifting to up-skill non-mental health professionals located within disaster-affected communities, including volunteers, greatly increases the potential reach and cost-effectiveness of the program. Pilot studies have shown it to be safe, acceptable, replicable, efficacious, and able to be culturally adapted and evaluated [62,63]. There are currently a number of RCTs underway to further test its efficacy after disaster and trauma.

Clinical guidelines typically recommend the use of EBTs for mental health disorders arising from EWEs, usually delivered by qualified mental health professionals. For example, Trauma-Focused Cognitive-Behavioral Therapy (TF-CBT) [64,65], Cognitive Processing Therapy (CPT) [66,67], Narrative Exposure Therapy [68,69], Cognitive Behavioral Therapy—Post Disaster (CBT-PD) [70], and Eye Movement Desensitization and Reprocessing (EMDR [71] have been found to be effective in the treatment of posttraumatic stress disorder in disaster survivors. Cognitive-Behavioral Intervention for Trauma in Schools (CBITS) [65,72], the web-based Bounce Back Now (BBN) [73,74], and other school-based interventions such as Enhancing Resiliency among Students Experiencing Stress—Sri Lanka (ES-SL), [75] Catastrophic Stress Intervention (CSI) [76], and Loss and Survival Team (LAST) [77,78] have been found to be effective in treating posttraumatic stress, depression, and anxiety in children and adolescents exposed to extreme weather events. All of these school-based interventions incorporate the principles and practice of cognitive-behavioral therapy and narrative exposure therapy [79]. Although each of these interventions was individually rated as 2 or 3, collectively, they earned a rating of 1. Likewise, cognitive behavioral-based interventions are recommended for depression and anxiety disorders [80,81] in survivors of extreme weather events [41,82].

Unfortunately, the evidence supporting the effectiveness of these interventions with survivors of acute climate-related events is limited, especially in low resource settings that have few trained clinicians and mental health facilities [10]. Unlike some of the indicated interventions like PM+ and SOLAR that have been evaluated in low-resource settings, many of these more intensive evidence-based treatments cannot simply be transferred or extrapolated from high-resource to low-resource settings without substantial adaptation and evaluation of effectiveness and acceptability. Research is needed to evaluate the effectiveness of existing EBTs for addressing the post-disaster mental health needs in these settings and to devise strategies for successfully implementing and sustaining these EBTs [83]. An important cross-cutting dimension in these efforts will be the adaptation of interventions and implementation strategies to ensure they are culturally appropriate and responsive to socioeconomic and cultural differences in exposure to acute forms of climate change and their mental health impacts [36]. For instance, teams of volunteers trained to provide a culturally-tailored form of psychosocial support known as “kokoro-no care” to survivors of the Great East Japan Earthquake in 2011 have become a standard feature of post-disaster mental health and psychosocial support in Japan [84,85]. This form of support is designed to provide universal services such as listening, counseling, lectures, recreation, group activities, and relaxation techniques by non-specialists that increase mental health literacy and reduce the stigma associated with mental health problems.

### 3.3. Development and Training of Disaster Responders

Plans for responding to EWEs typically include recommendations for the training of personnel to address the mental health needs of communities impacted by EWEs and other natural disasters and forms of mass trauma [30,31,33]. For instance, the US Substance Abuse and Mental Health Services Administration (SAMHSA) has identified 34 online disaster behavioral health training opportunities skills pertaining to psychological disaster response [86]. The NATO Joint Medical Committee [33] organized training requirements for incident response commanders, managers, and professional staffs into a four-tier model of stepped care services delivery. The levels are:

- Tier 1: General training in core knowledge, attitudes, and skills (required by all responders and professionals who work in the context of disasters and major incidents);
- Tier 2: More advanced training for those who deliver psychological first aid, basic psychological therapies, and assessment of people who may require more specialized mental healthcare;
- Tier 3: Specialist training required by staff who deliver the functions of Levels 3 and 4 in the model of care in which a personal approach to particular people’s needs is based on the assessment of their needs. This includes training to supervise staff whose work includes delivering psychosocial care at Levels 1 and 2 in a stepped care model;
- Tier 4: Advanced specialist training for professionals who are appointed to provide advice to major incident response commanders at strategic, operational, and tactical levels. These appointments require not only disaster-related training in psychosocial and mental healthcare but also training in major incident management, consultative skills, and selected aspects of strategic leadership and management (pp. 1–109, [33]).

Tier 2 training would also include training in brief low-intensity interventions such as PFA, PM+, SPR, or SOLAR.

An effective and sustainable stepped care approach to mental health service delivery after a natural disaster will inevitably require the recruitment and training of non-mental health specialists, who are members of affected populations, with an understanding of mental health’s social and cultural context and service delivery [3,13]. These non-specialists include school personnel trained to deliver Tier 3 interventions like CBITS [72] and local residents trained as community health workers to deliver less-intensive Tier 1 and 2 services like PFA or SPR [87,88,89].

As noted earlier, task-shifting and training of non-mental health professionals is critical to service delivery in low-and middle-income countries and rural areas of high-income countries where the number of mental health specialists is quite limited. However, limited resources for the delivery of mental health services also necessitate a greater emphasis on the prevention of psychiatric disorders and behavioral health problems and the promotion of positive public mental health initiatives. A focus on mental health promotion is a key ingredient of sustainable development and should be incorporated into the training and support of specialists and non-specialists.

### 3.4. Needs Assessments

Disaster preparedness guidelines and protocols also include assessments of available resources and at-risk populations in need of appropriate levels of mental health services in the aftermath of an EWE. Such protocols include the mapping and monitoring of psychosocial resources and skills within communities [30,31,33,40] and the use of resources like the Disaster Psychosocial Assessment and Surveillance Toolkit (Disaster-PAST) to improve disaster preparedness and response and enhance community recovery [90]. Disaster preparedness protocols should also include procedures for identifying individuals and communities that are most underserved and most susceptible to the mental health impacts of acute forms of climate change [30,31,33,40]. These include women, children, and youth, persons with disabilities, poor people, migrants, older persons, individuals with a history of mental health problems, indigenous peoples, and individuals with little professional or social support [2,4,5,10,11,91,92]. Residents of low and middle-income countries and low resource areas of high-income countries (e.g., rural areas, poor urban neighborhoods) are especially vulnerable to climate-related mental health problems due to their increased exposure to EWEs and other traumatic events (e.g., civil conflicts), high levels of poverty or economic adversity, and lack of access to services [1,2,13,91,92]. Geographic Information Systems can enable mental health providers to more effectively locate specific locations and groups at risk for elevated mental health impacts and to prioritize resources and personnel to deliver care in the aftermath of an acute EWE [11].

### 3.5. Mental Health Services for Sub-Acute Events

In addition to the indicated prevention and standard treatment services needed to respond to acute climate change-related events described above, specialized services will be required to prevent or treat at-risk populations. These may include individuals with pre-existing psychiatric conditions who have serious mental health problems during heatwaves [17], and farmers in drought-affected regions at risk for suicide [18,19]. For instance, The Community Response to Eliminating Suicide (CORES) program is a community-based and gatekeeper education model that aims to build and empower communities to take ownership of suicide prevention strategies, increase the individual community member’s interpersonal skills and awareness of suicide risks, and build peer support and awareness of suicide prevention support services within the community itself [93]. Mental Health First Aid (MHFA) in a psychosocial intervention designed to provide non-mental health specialists with evidence-based resources to provide help and appropriate referrals to people experiencing a mental health crisis (such as an episode of acute psychosis) or an ongoing mental health problem (such as depression) [94]. The efficacy of MHFA as a training tool was demonstrated in a randomized controlled trial in rural New South Wales [95]; to date, however, there is no evidence that it has resulted in a significant reduction of mental health problems or crises.

Responding to the mental health consequences of sub-acute events will also require strategies rooted in both mitigation and adaptation [10]. Mental health professionals are well-positioned to advocate for policies and programs that mitigate the likelihood and the magnitude of sub-acute and long-lasting forms of climate change [3,4,5,6,10], including the guidelines and interventions recommended for responding to acute forms of climate change described above. Many of these guidelines and interventions will undoubtedly require forms of adaptation to address the secondary impacts of sub-acute forms of climate change, such as threats to economic livelihood, health and well-being, population displacement, environmental degradation, and civil conflict, as well as the populations most vulnerable to such impacts. For instance, persistent flooding and coastal erosion resulting from sea-level rise and more severe EWEs will necessitate re-locating entire communities to safer and more environmentally sustainable inland areas [10]. Such relocation will result in an increased need for mental health services arising from migration while reducing the risk of mental health impacts due to the stress of living in degraded environments. Mental health service providers will increasingly rely on public health education to address the stress associated with the increasing threats of vector-borne illnesses due to climate change [10]. Mitigation of the mental health consequences of civil conflicts and interpersonal violence will also require increased attention to underlying risk factors such as poverty and socioeconomic disparities [25].

### 3.6. Mental Health Services for Long-Lasting Events

In addition to the strategies for delivering services for acute and sub-acute events, strategies for delivering universal services to address the mental health impacts of long-lasting climate-related events are required. Younger generations are especially vulnerable to these impacts. A survey commissioned by the Washington Post and Kaiser Family Foundation of American Youth found that more than 70% believe they will be harmed by climate change. About 57% of teens and 68% of young adults interviewed reported that climate change makes them feel afraid [96].

One potential strategy for addressing these impacts is the implementation of universal evidence-based risk communication interventions that address the existing and potential threat of climate change. Such interventions can be targeted to populations where climate adaptation efforts are most needed [97]. There are several evidence-based strategies for communicating risks that can be employed in educating the public regarding the long-lasting risks associated with climate change and motivating behavior to mitigate those risks [98,99]. However, the challenge lies in not merely increasing awareness of the risks associated with climate change, but in also managing feelings of stress and anxiety. Mah and colleagues [100] call for attending to differences in the kinds of stress associated with climate change, individual differences in stress response, and the influence of community resilience on individual coping when implementing these evidence-based communication strategies.

Another universal strategy is to promote the mental health benefits of environmental conservation. Consistent with the principles and practice of ecotherapy [101,102], assuming responsibility for protecting the environment through deliberate action has been linked to addressing the “psychoterratic” syndromes of ecoanxiety, ecoparalysis, and solastalgia [2,5,10,35]. The biodiversity in natural environments that are preserved through conservation efforts, and engagement in climate change mitigation efforts both have been demonstrated to result in positive effects on mood, attention, and cognition [10,36,103,104]. However, to date, there have been no controlled trials of the effectiveness of ecotherapy-based interventions in reducing these psychoterratic syndromes.

A third universal strategy is to promote elements of positive mental health impacts of climate change. Awareness of the threat of climate change to human health and well-being may also inspire and support psychosocial resilience, is based on evidence of individual and community responses to extreme weather events with compassion, altruism, and posttraumatic growth [10,105]. However, research is required to determine whether the positive responses to acute events can be effectively translated into evidence-based practices that enable individuals and communities to effectively adapt to long-lasting changes in the environment, given the greater difficulty in mobilizing responses to existing and potential threats that are experienced slowly and indirectly, compared to threats that are experienced immediately and personally [106].

Nevertheless, a good part of the solutions proposed for the impacts of long-lasting climate-related events, will require broader and more varied political institutions and programs (such as employment, housing, urban development, economic or environmental policies, among others). Although mental health services can play a key role in mitigating the impacts on people’s health, in preventive terms public policies will surely be more effective in reducing the need for such services.

## 4. Conclusions

Mental health services providers are increasingly called upon to address the consequences of acute, sub-acute, and long-lasting forms of climate change. These consequences may be the result of direct exposure to changes in the physical environment or to secondary impacts such as economic losses, illness and injury, forced migration, and civil conflict. Some of the mental health impacts and services employed in response to these impacts will crosscut all three event types, and some will be specific to each type of event. The services that are currently utilized or likely to be developed in response to acute and extreme weather events will likely play an important role in responding to the mental health consequences of longer duration events. This review presented a continuum of services designed to prevent and treat mental and behavioral health problems associated with acute, sub-acute, and long-lasting changes in the physical environment due to global climate change. However, as these problems manifest themselves for longer periods of time, impacting larger segments of the population, their solutions will inevitably rely upon the implementation of policies and programs designed to mitigate the changes in the physical environment and to promote the health and well-being of the global community. Examples of such policies and programs include planned relocation of individual climate migrants and entire communities, sustainable social and economic development, decarbonization of the economy, public health education, violence prevention, risk communication, personal engagement in environmental conservation, and promotion of positive psychological growth and individual and community resilience.

## Figures and Tables

**Table 1:** Inclusion and exclusion criteria.

Inclusion Criteria Item	Description	Justification
Population	Persons aged 5–85	Age limits were based on two considerations: (1) populations exposed to climate change, and therefore, at risk for adverse mental health impacts include all age groups [1,2,3,4,5,6,7,8,9,10,11,12,13,14,15,16,17,18,19,20]; and (2) target populations of interventions designed to prevent and treat these outcomes including those targeting school-aged youth, adults, and older adults.
Intervention	Universal, selective, and indicated interventions designed to prevent or mitigate symptoms of posttraumatic stress, depression, anxiety, or any forms of psychosocial dysfunction. Interventions designed to treat individuals meeting diagnostic criteria for PTSD, depressive, or anxiety disorders.	Selection of interventions was based on the classification of interventions provided by the National Academy of Sciences [28] and guidelines and recommendations provided by international bodies for addressing mental health outcomes of disasters and other emergencies. These included the World Health Organization [30], United Nations [31,32], and North Atlantic Treaty Organization (NATO) Joint Medical Committee [33]
Comparisons and outcomes	All reported assessments of mental health outcomes using validated measures	Assessments of efficacy or effectiveness require the use of standardized and validated measures of mental health status to ensure results are valid, replicable, and generalizable.
Study design	Qualitative, mixed methods and quantitative studies such as descriptive studies, research case studies, pre-post trials, RCTs, and evaluation studies	Few interventions have been designed specifically to address the mental health impacts of climate change; hence a decision was made to include exploratory investigations of potential interventions as well as investigations of interventions developed to prevent and treat mental health problems associated with other traumatic events (i.e., civil conflicts, terrorism, manmade disasters, earthquakes).
Articles	English-language articles published in academic journals that follow a peer-review publication process	Although this review did not assess the risk of bias, it did seek to identify studies that adhered to the World Medical Association Declaration of Helsinki—Ethical Principles for Medical Research Involving Human Subjects. The selection of peer-reviewed publications helped to ensure a baseline for evaluation of the quality of scientific evidence.
Publication date	2000–2020	A preliminary review of literature reviewed no peer-reviewed publications on the topic appearing prior to 2000.

**Table 2:** Level of proposed mental health services delivery by type of climate-related event.

IOM Service Categories and Approaches		Climate-Related Events		
		Acute	Subacute	Long-Lasting
**Prevention**	Universal	PFA IMHD Mapping Tier 1–2 training SFP	MHFA CORES Advocacy Adaptation Mapping Tier 1–2 training	Risk communication Ecotherapy Psychosocial resilience Tier 1–2 training
	Selective	SFP Tier 1–2 training	Suicide prevention Heat exposure interventions Tier 1–2 training	
	Indicated	Tier 1–3 training PM+ SPR SOLAR	Tier 1–3 training PM+ SPR SOLAR	Tier 1–3 training PM+ SPR SOLAR
**Treatment**	Standard	CBITS BBN TF-CBT CPT CBT-PD NET EMDR CBT for Anxiety and Depression Tier 3-4 training	CBITS BBN TF-CBT (conflict) CPT (conflict) CBT-PD (conflict) NET (conflict) EMDR (conflict) CBT for Anxiety and Depression Tier 3-4 training	CBITS BBN CBT for Anxiety and Depression Tier 3–4 training

Legend: BBN = bounce back now, CBITS = Cognitive-behavioral intervention for trauma in schools, CBT-PD = cognitive-behavioral therapy-post disaster, CORES = community response to eliminating suicide, CPT = cognitive processing therapy, EMDR = eye movement desensitization and reprocessing, IMHD = integrated mental health and disaster, MHFA = mental health first aid, NET = narrative exposure therapy, PFA = psychological first aid, PM+ = problem management plus, SOLAR = skills for life adjustment and resilience, SFP = Strengthening families program, SPR = skills for psychological recovery, tier 1 training = general training in core knowledge, attitudes and skills; tier 2 training = more advanced training for those who deliver PFA, basic psychological therapies and assessment; Tier 3 training = specialist training; tier 4 training = advanced specialist training. TF-CBT = trauma-focused cognitive behavioral therapy. Conflict refers to use in conflict settings.

**Table 3:** Level of mental health services delivery by type of climate-related event.

Intervention	Ratings of Scientific Evidence					
	Acute Events	Refs	Sub-Acute Events	Refs	Long-Term Events	Refs
Universal and selective interventions						
Psychological first aid	2 *	Kang, J.Y. et al., 2020; Sijbrandij, M. et al., 2020	NR		NR	
Mental health first aid	NR		2 *	Sartore, G.M. et al., 2008; Jorm, A.F. et al., 2004	NR	
Ecotherapy	NR		NR		NR	
Strengthening families program	NR		NR		NR	
Integrated mental health and disaster preparedness	2	James, L.E. et al., 2020	NR		NR	
Community response to eliminating suicide	NR		NR		NR	
Indicated interventions						
Problem management plus	NR		NR		NR	
Skills for psychological recovery	3	Bisson, J.I. et al., 2010	NR		NR	
SOLAR	3	Gibson, K. et al., 2019, O’Donnell, M.L. et al., 2020	NR		NR	
Treatment interventions						
School-based interventions	1	Jaycox, L.H. et al., 2010; Jaycox, L., 2004; Ruggiero, K.J. et al., 2015; Gilmore, A.K. et al., 2018; Berger, R. et al., 2009; Chemtob, C.M. et al., 2002; Salloum, A. et al., 2008; Salloum, A. et al., 2012; Hardin, S.B. et al., 2002	NR		NR	
Trauma focused-cognitive behavioral therapy	2	Jaycox, L.H. et al., 2010	NR		NR	
Cognitive-behavioral therapy-Post disaster	3	Hamblen, J.L. et al., 2009	NR		NR	
Cognitive processing therapy	NR		NR		NR	
Cognitive-behavioral therapy for depression or anxiety	2	Bisson, J.I. et al., 2010; American Psychological Association Guideline Development Panel for the Treatment of Depressive Disorders, 2019	NR			
Narrative exposure therapy	2	Catani, C. et al., 2009; Crombach, A. et al., 2018	NR		NR	
Eye movement desensitization and reprocessing	2	Chemtob, C.M. et al., 2002	NR		NR	

Legend: Ratings of scientific evidence 1 = well supported, 2 = supported, 3 = promising, 4 = potential, NR Not rated. * Supported by scientific evidence as a training tool but not as a therapeutic intervention.
